# Mechanisms of acquired resistance to osimertinib in EGFR T790M-Positive lung adenocarcinoma

**DOI:** 10.3389/fgene.2026.1827187

**Published:** 2026-09-04

**Authors:** Kefan Cui, Renjie Zhu, Jingmeng Li

**Affiliations:** 1 Department of Thoracic Surgery, Songshan General Hospital, Chongqing, China; 2 Department of Thoracic Surgery, The People’s Hospital of Chongqing Liangjiang New Area, Chongqing, China

**Keywords:** C797S mutation, epidermal growth factor receptor, lung adenocarcinoma, osimertinib, resistance mechanisms, T790M mutation

## Abstract

**Background:**

Third-generation epidermal growth factor receptor tyrosine kinase inhibitor osimertinib serves as the gold standard therapy for treating NSCLC patients harboring EGFR T790M mutations. The clinical utility of this agent, however, faces considerable constraints due to the unavoidable emergence of acquired resistance mechanisms. Understanding the molecular basis of osimertinib resistance holds paramount importance for determining optimal follow-up treatment approaches.

**Methods:**

Clinical information from 86 lung adenocarcinoma patients carrying EGFR T790M mutations who showed disease advancement following osimertinib therapy was examined retrospectively, spanning the period from January 2018 through December 2023. Genomic characterization was conducted via next-generation sequencing on tissue or liquid biopsy specimens collected after resistance developed. Protein expression alterations were assessed through immunohistochemical staining, while critical resistance pathways underwent validation using cell line models.

**Results:**

Among the 86-patient cohort, the median duration before disease progression reached 14.2 months. Genomic characterization identified these predominant resistance pathways: C797S mutations in EGFR (23.3%), amplification of MET (15.1%), amplification of HER2 (8.1%), mutations in PIK3CA (7.0%), transformation to small cell lung cancer (9.3%), and epithelial-mesenchymal transition (12.8%). Concurrent presence of multiple resistance mechanisms was detected in 24.4% of the patient population. Within the C797S mutation subset, 65.0% exhibited C797S/T790M in cis arrangement, 30.0% demonstrated trans arrangement, and 5.0% showed mixed configurations. Laboratory validation established that MET amplification confers resistance via bypass activation of both ERK and AKT signaling cascades. The poorest clinical outcomes were observed among patients undergoing histological transformation (median overall survival from confirmed progression: 8.3 months).

**Conclusion:**

Remarkable heterogeneity characterizes the resistance mechanisms emerging against osimertinib in EGFR T790M-positive lung adenocarcinoma, with EGFR secondary mutations, bypass signaling pathway activation, and histological transformation representing the primary categories. Detection of specific resistance mechanisms enables tailored subsequent therapeutic approaches, with potential outcome improvements achievable through combination strategies incorporating targeted agents or immunotherapy.

## Introduction

Worldwide, lung cancer continues as the principal contributor to cancer mortality, with NSCLC representing roughly 85% of total diagnoses (Sung et al., 2021; Ettinger et al., 2022). Lung adenocarcinoma constitutes the predominant histological variant among NSCLC cases. Activating alterations within the EGFR gene occur in roughly 10%–15% of patients of Caucasian descent and 40%–50% of patients of Asian ancestry who have lung adenocarcinoma (Shi et al., 2014; Zhang et al., 2016; Midha et al., 2015), establishing EGFR as an essential target in precision medicine approaches.

Treatment paradigms for EGFR-mutant NSCLC underwent revolutionary changes following the introduction of EGFR TKIs. When compared to platinum-containing chemotherapy regimens, both first-generation agents (gefitinib, erlotinib) and second-generation agents (afatinib, dacomitinib) yielded substantial clinical advantages (Mok et al., 2009; Park et al., 2016). Nonetheless, initial remarkable therapeutic responses notwithstanding, acquired resistance invariably manifests within 9–14 months across essentially all treated patients (Yu et al., 2013). Representing roughly 50%–60% of acquired resistance instances to first- and second-generation TKI therapies, the EGFR T790M gatekeeper mutation presented a substantial therapeutic obstacle (Oxnard et al., 2011).

Specifically engineered to inhibit both EGFR-sensitizing alterations and the T790M resistance alteration while preserving wild-type EGFR function, osimertinib represents a third-generation irreversible EGFR TKI (Cross et al., 2014). Among T790M-positive patients experiencing progression following earlier EGFR TKI treatment, clinical investigations established osimertinib’s superior effectiveness, with median PFS approximating 10 months (Mok et al., 2017). Additionally, for EGFR-mutant advanced NSCLC, osimertinib has emerged as the favored initial therapeutic option, demonstrating marked PFS improvements and enhanced central nervous system disease management relative to first-generation TKIs (Soria et al., 2018). Nevertheless, paralleling earlier-generation TKIs, osimertinib encounters inevitable acquired resistance development, thereby restricting its sustained clinical utility.

Developing rational follow-up treatment approaches necessitates comprehensive understanding of the molecular basis underlying osimertinib resistance. Earlier investigations have revealed various potential resistance pathways, encompassing tertiary EGFR alterations (notably C797S) (Thress et al., 2015; Ramalingam et al., 2018), bypass signaling pathway activation, and histological transformation (Sequist et al., 2011). Nonetheless, comprehensive resistance mechanism analyses across extensive patient populations remain scarce, leaving the comparative frequencies and clinical significance of distinct resistance mechanisms incompletely characterized.

Through this investigation, we sought to comprehensively delineate the molecular architecture of acquired osimertinib resistance among patients harboring EGFR T790M-positive lung adenocarcinoma. Employing comprehensive genomic characterization via next-generation sequencing alongside *in vitro* functional validation, our objectives included determining the resistance mechanism spectrum, establishing their comparative prevalence, and evaluating their correlated clinical outcomes. Critical understanding of osimertinib resistance heterogeneity emerges from our observations, potentially guiding precision therapeutic strategy development for patients manifesting disease advancement during osimertinib treatment.

Unlike prior single-institution series and pooled analyses that predominantly catalogued resistance mechanism types, the present study advances the field in three ways: (1) it provides direct quantitative comparison of mechanism frequencies in a well-characterized real-world cohort, revealing that bypass pathway activation collectively accounts for 30.2% of resistance events and rivals on-target C797S in clinical importance; (2) it documents spatial and temporal heterogeneity through multi-site and sequential biopsies, underscoring the inadequacy of single-site profiling; and (3) it links specific mechanism categories to distinct survival strata, enabling evidence-based post-progression treatment stratification. The present analysis also incorporates recent advances in circulating tumor DNA (ctDNA)-based resistance profiling, FLAURA long-term follow-up data, and combination therapeutic strategies targeting bypass pathways, thereby contextualizing findings within the rapidly evolving landscape of EGFR-targeted therapy (Papadimitrak et al., 2020; Ramalingam et al., 2020; Engelman et al., 2007). The integration of multivariable predictive models has further enriched clinical decision-making frameworks in oncology; recent work has demonstrated the feasibility of combining clinical and molecular variables into validated models that stratify patients by post-treatment complication risk, an approach increasingly applicable to post-progression management in EGFR-mutant NSCLC (Zhu et al., 2025).

## Methods

### Patient selection and clinical data collection

Our retrospective analysis encompassed 86 patients diagnosed with EGFR T790M-positive lung adenocarcinoma who manifested disease advancement after undergoing osimertinib treatment at our medical center during the timeframe spanning January 2018 to December 2023. Histologically verified lung adenocarcinoma with documented EGFR-activating alterations (exon 19 deletion or L858R point mutation) plus acquired T790M mutation identified before osimertinib commencement characterized all enrolled patients. Eligibility criteria mandated osimertinib administration for no less than 8 weeks with achievement of complete response, partial response, or stable disease per Response Evaluation Criteria in Solid Tumors (RECIST) version 1.1 preceding progressive disease development. Electronic medical record extraction provided clinical information encompassing patient demographics, smoking background, baseline Eastern Cooperative Oncology Group (ECOG) performance status, therapeutic history, radiological responses, progression-free survival, and overall survival. Radiological evaluation or clinical deterioration determined disease progression. Institutional ethics committee approval was secured for the study. Due to the retrospective nature of the study, the requirement for informed consent was waived.

### Sample collection and processing

Following osimertinib progression verification, molecular profiling to determine resistance mechanisms involved obtaining tissue biopsies or liquid biopsies. Depending on progressive lesion accessibility, tissue specimen acquisition occurred through CT-guided percutaneous biopsy, bronchoscopic biopsy, or surgical resection procedures. Immediate snap-freezing in liquid nitrogen or fixation in 10% neutral-buffered formalin for subsequent paraffin embedding processed fresh tumor tissues. When tissue biopsy proved infeasible due to inaccessible lesions or compromised performance status, liquid biopsy procedures involved collecting 10–15 mL peripheral blood in cell-free DNA collection tubes. Within 2 h of blood collection, plasma separation occurred via a two-step centrifugation protocol with −80 °C storage until analysis. Experienced pathologists evaluated tumor content in tissue specimens, with genomic analysis restricted to samples exceeding 20% tumor cellularity.

### Next-generation sequencing and genomic analysis

The QIAamp DNA FFPE Tissue Kit following manufacturer’s protocol extracted genomic DNA from formalin-fixed paraffin-embedded tissue specimens. Plasma-derived circulating cell-free DNA isolation for liquid biopsy specimens utilized the QIAamp Circulating Nucleic Acid Kit. Qubit fluorometer assessment determined DNA concentration and quality, with fragment size distribution analysis performed. A customized panel encompassing 520 cancer-associated genes, including complete exons of EGFR, MET, HER2, BRAF, PIK3CA, KRAS, ALK, ROS1, RET, plus additional genes commonly altered in lung cancer, enabled targeted next-generation sequencing. Hybrid capture-based target enrichment conducted library preparation, with Illumina NovaSeq 6000 platform sequencing achieving target coverage depth of 1000× for tissue specimens and 3000× for liquid biopsy specimens. Bioinformatic processing incorporated sequence alignment to human reference genome (hg19), established pipeline-based variant calling, and annotation of single nucleotide variants, insertions/deletions, copy number alterations, and gene fusions. Among patients carrying EGFR C797S mutations, cis or trans configuration determination relative to T790M employed sequencing read analysis containing both mutations or allele-specific PCR when required. MET amplification was defined as a MET/CEP7 ratio ≥2.2 or absolute MET copy number ≥6 signals/cell by fluorescence *in situ* hybridization (FISH), consistent with established clinical trial criteria. HER2 amplification was defined as a HER2/CEP17 ratio ≥2.0 or average HER2 copy number ≥6.0 signals/cell by FISH, with protein overexpression confirmed by immunohistochemistry (3+ intensity). For single nucleotide variants and small insertions/deletions, minimum variant allele frequency (VAF) thresholds of 1.0% for tissue specimens and 0.5% for liquid biopsy specimens were applied. Cis configuration of C797S and T790M was confirmed when both mutations co-occurred within the same sequencing read or read pair; trans configuration was assigned when mutations appeared on separate alleles based on allele frequency imbalance, with allele-specific PCR performed for confirmation in ambiguous cases.

### Immunohistochemical staining and histological assessment

Formalin-fixed paraffin-embedded tissue sample 4-μm sections underwent immunohistochemical staining to assess protein expression alterations and identify histological transformation. Deparaffinization and rehydration preceded antigen retrieval using citrate buffer (pH 6.0) in a pressure cooker. Blocking of endogenous peroxidase activity employed 3% hydrogen peroxide, with normal serum preventing nonspecific binding. Overnight incubation at 4 °C with primary antibodies against thyroid transcription factor-1 (TTF-1), synaptophysin, chromogranin A, CD56, E-cadherin, vimentin, and epithelial-mesenchymal transition markers occurred on sections. The EnVision + system with diaminobenzidine as chromogen accomplished detection, followed by hematoxylin counterstaining. Two independent pathologists blinded to clinical information assessed staining outcomes. Morphological features including small cell size, scant cytoplasm, finely granular chromatin, and positive neuroendocrine marker staining (synaptophysin, chromogranin A, or CD56) with absent TTF-1 or weak TTF-1 expression established small cell lung cancer transformation diagnosis. Loss of E-cadherin expression combined with vimentin expression gain and characteristic spindle cell morphology defined epithelial-mesenchymal transition.

### Cell culture and in vitro resistance models

Validation of identified resistance mechanisms employed EGFR-mutant lung adenocarcinoma cell lines encompassing PC9 (exon 19 deletion) and H1975 (L858R/T790M). RPMI-1640 medium supplemented with 10% fetal bovine serum cultured cells, with maintenance at 37 °C in a humidified atmosphere containing 5% CO2. Chronic exposure to progressively increasing osimertinib concentrations over 6–8 months generated osimertinib-resistant cell lines. MET expression vector transfection of parental cells or hepatocyte growth factor treatment to activate MET signaling modeled MET amplification-mediated resistance. After 72 h of drug treatment, CCK-8 assay assessed cell viability, with nonlinear regression analysis calculating IC50 values. Western blotting evaluated downstream signaling pathway activation status including ERK and AKT. Specifically, RIPA buffer containing protease and phosphatase inhibitors lysed cells, with equal protein amounts undergoing SDS-PAGE separation, PVDF membrane transfer, and probing with specific antibodies against phospho-ERK, total ERK, phospho-AKT, total AKT, and GAPDH as loading control. All functional experiments were conducted in three independent biological replicates performed in technical triplicate. IC50 values from CCK-8 assays are reported as mean ± SD with 95% confidence intervals. Western blot band intensities were quantified by densitometric analysis normalized to GAPDH using ImageJ software, and inter-group comparisons were performed by unpaired two-tailed t-test. It is acknowledged that MET overexpression and HGF stimulation do not fully recapitulate the transcriptional and chromosomal context of genuine genomic amplification; this limitation is explicitly addressed in the Discussion section.

### Statistical analysis

Osimertinib initiation to radiological or clinical disease progression or death from any cause defined progression-free survival. Overall survival for the full cohort was measured from osimertinib initiation to death from any cause; overall survival for resistance mechanism subgroups was uniformly measured from the date of confirmed radiological or clinical progression on osimertinib to death from any cause. Kaplan-Meier methodology constructed survival curves, with log-rank test comparing group differences. Chi-square test or Fisher’s exact test as appropriate compared categorical variables. Median with interquartile range expressed continuous variables, with Mann-Whitney U test or Kruskal–Wallis test providing comparisons. Multivariable Cox proportional hazards regression analysis determined independent prognostic factors associated with overall survival. This analytical framework—combining Kaplan-Meier survival estimation, log-rank testing, and multivariable Cox proportional hazards regression—has been similarly applied in recent prognostic studies across critical illness contexts to identify independent predictors of clinical outcomes (Chen et al., 2025). Variables were pre-selected based on clinical relevance and prior literature: age (≥65 vs. <65 years), sex, ECOG performance status (0–1 vs. 2–3), osimertinib treatment line (second-line vs. third-line or later), resistance mechanism category (histological transformation, multiple concurrent mechanisms, or single mechanism), and receipt of post-progression targeted therapy. To address potential treatment contamination bias, post-progression targeted therapy was incorporated as a covariate; a sensitivity analysis restricted to patients who did not receive subsequent targeted therapy yielded directionally consistent results. Survival analyses were not performed for resistance subgroups with fewer than five patients (BRAF V600E, n = 3; RET fusion, n = 1); these cases are described descriptively only. All hazard ratios (HR) and 95% confidence intervals (CI) are systematically reported. Two-sided P-values below 0.05 were considered statistically significant. SPSS version 26.0 and R software version 4.2.0 conducted statistical analyses.

## Results

### Patient characteristics and clinical outcomes

This study enrolled 86 patients diagnosed with EGFR T790M-positive lung adenocarcinoma who developed acquired osimertinib resistance. Patient median age reached 61 years (range: 38–79 years), with female representation at 52.3%. Never-smokers comprised the majority (73.3%), while 58.1% demonstrated ECOG performance status of 0–1 when osimertinib commenced. Within the cohort, EGFR exon 19 deletions characterized 62.8%, whereas L858R point mutations as the initial sensitizing alteration affected 37.2%. T790M mutation development following first- or second-generation EGFR TKI therapy preceded subsequent osimertinib receipt as second-line or later treatment across all patients. On osimertinib, median progression-free survival achieved 14.2 months (95% confidence interval: 12.8–15.9 months), representing notably extended duration compared to historical data with earlier-generation TKIs. Data cutoff documented 68 patient deaths (79.1%), with median overall survival from osimertinib initiation measuring 22.4 months. Successful molecular profiling at progression encompassed all 86 patients, with tissue biopsy performance in 67 patients (77.9%) and liquid biopsy performance in 19 patients (22.1%) (Table 1). A patient screening flowchart detailing the eligibility process is provided in Supplementary Figure S1. Stratified analyses by biopsy type demonstrated a resistance mechanism detection rate of 92.5% in tissue biopsy patients versus 68.4% in liquid biopsy patients (P = 0.002, Fisher’s exact test); stratified results by biopsy modality are presented in Supplementary Table S1. Critically, MET amplification was detected exclusively in tissue biopsy specimens and in none of the 19 liquid biopsy cases, consistent with the known limited sensitivity of cell-free DNA-based platforms for copy number alterations. Similarly, HER2 amplification was confirmed only in tissue specimens. These findings have direct clinical implications: when copy number-driven bypass mechanisms are clinically suspected, tissue biopsy should be prioritized over liquid biopsy for post-progression molecular profiling. Complete stratified results by biopsy modality, including detection rates for each individual resistance mechanism, are presented in Supplementary Table S1.

**TABLE 1:** Baseline patient characteristics and clinical outcomes.

Characteristic	Total (n = 86)	Exon 19 deletion (n = 54)	L858R mutation (n = 32)	Statistical value	P-value
Demographics					
Age, median (range), years	61 (38–79)	60 (38–77)	63 (42–79)	Z = 1.014	0.312
Age ≥65 years, n (%)	32 (37.2)	19 (35.2)	13 (40.6)	χ^2^ = 0.261	0.609
Female, n (%)	45 (52.3)	30 (55.6)	15 (46.9)	χ^2^ = 0.636	0.426
Smoking status, n (%)	​	​	​	χ^2^ = 0.413	0.521
Never-smoker	63 (73.3)	41 (75.9)	22 (68.8)	​	​
Former/Current smoker	23 (26.7)	13 (24.1)	10 (31.2)	​	​
ECOG PS at osimertinib initiation, n (%)	​	​	​	χ^2^ = 0.162	0.687
0–1	50 (58.1)	32 (59.3)	18 (56.2)	​	​
2–3	36 (41.9)	22 (40.7)	14 (43.8)	​	​
EGFR mutation type, n (%)					
Exon 19 deletion	54 (62.8)	54 (100)	0 (0)	NA	NA
L858R point mutation	32 (37.2)	0 (0)	32 (100)	NA	NA
Treatment line for osimertinib, n (%)	​	​	​	χ^2^ = 0.106	0.745
Second-line	58 (67.4)	37 (68.5)	21 (65.6)	​	​
Third-line or later	28 (32.6)	17 (31.5)	11 (34.4)	​	​
Biopsy type at progression, n (%)	​	​	​	χ^2^ = 0.018	0.892
Tissue biopsy	67 (77.9)	42 (77.8)	25 (78.1)	​	​
Liquid biopsy	19 (22.1)	12 (22.2)	7 (21.9)	​	​
Clinical outcomes					
PFS, median (95% CI), months	14.2 (12.8–15.9)	15.1 (13.2–17.3)	12.8 (10.9–14.6)	Log-rank χ^2^ = 2.935	0.087
OS from osimertinib initiation, median (95% CI), months	22.4 (19.7–25.8)	23.6 (20.1–27.4)	20.3 (16.8–24.1)	Log-rank χ^2^ = 1.996	0.156
Deaths at data cutoff, n (%)	68 (79.1)	41 (75.9)	27 (84.4)	χ^2^ = 0.909	0.340

ECOG PS, eastern cooperative oncology group performance status; CI, confidence interval; PFS, progression-free survival; OS, overall survival; NA, not applicable.

Statistical values: χ^2^ values from chi-square test or Fisher’s exact test for categorical variables; Z values from Mann-Whitney U test for continuous non-parametric data; Log-rank χ^2^ for survival analysis.

### Spectrum and distribution of resistance mechanisms

Comprehensive genomic profiling revealed a heterogeneous landscape of resistance mechanisms following osimertinib treatment. EGFR C797S mutation emerged as the most prevalent mechanism, identified in 20 patients (23.3% of the cohort), followed by MET amplification in 13 patients (15.1%). Additional bypass pathway activation was observed through HER2 amplification in seven patients (8.1%) and PIK3CA mutations in six patients (7.0%). Histological transformation represented another major category of resistance, with small cell lung cancer transformation detected in eight patients (9.3%) and epithelial-mesenchymal transition identified in 11 patients (12.8%). Other less common mechanisms included BRAF V600E mutation (3 patients, 3.5%), KRAS mutations (2 patients, 2.3%), and RET fusion (1 patient, 1.2%). Notably, no identifiable resistance mechanism was detected in nine patients (10.5%) despite comprehensive genomic profiling, suggesting the existence of additional uncharacterized resistance pathways or potential limitations in detection methodologies. The diverse spectrum of resistance mechanisms underscores the molecular complexity and adaptive capacity of tumors under selective pressure from targeted therapy (Table 2).

**TABLE 2:** Spectrum of resistance mechanisms following osimertinib treatment.

Resistance category	Specific mechanism	Number of patients	Percentage (%)
On-target resistance	EGFR C797S mutation	20	23.3
Bypass pathway activation	MET amplification	13	15.1
	HER2 amplification	7	8.1
	PIK3CA mutations	6	7.0
Histological transformation	Small cell lung cancer (SCLC) transformation	8	9.3
	Epithelial-mesenchymal transition (EMT)	11	12.8
Other bypass mechanisms	BRAF V600E mutation	3	3.5
	KRAS mutations	2	2.3
	RET fusion	1	1.2
Unknown mechanism	No identifiable resistance mechanism	9	10.5

Individual mechanism counts sum to more than 86 because 21 patients (24.4%) harbored multiple concurrent resistance mechanisms and are counted in each applicable category. Counts reflect mechanism occurrences rather than unique patients. Co-occurrence patterns among resistance mechanisms are illustrated in Supplementary Figure S2 (UpSet plot).

### Characteristics of EGFR C797S-Mediated resistance

**FIGURE 1:**
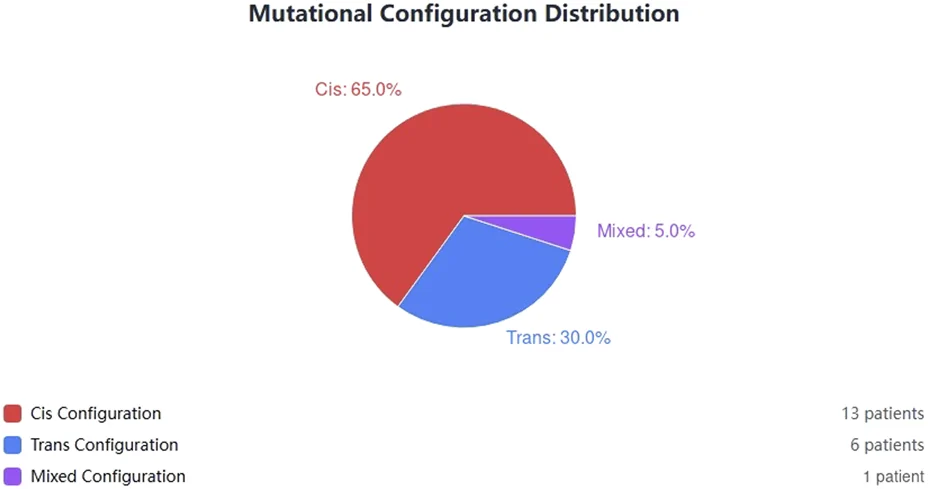
Distribution of EGFR C797S Mutational Configurations. Pie chart showing mutational configuration patterns in 20 osimertinib-resistant patient with C797S mutations. Cis configuration (both T790M and C797S on same allele): 65.0% (n = 13); Trans configuration (mutations on different alleles): 30.0% (n = 6); Mixed configuration: 5.0% (n = 1). The cis configuration confers resistance to both osimertinib and first-generation TKIs, while trans configuration may retain sensitivity to combination therapy.

Among the 20 patients harboring EGFR C797S mutations, detailed analysis of the mutational configuration revealed critical insights into the mechanism of resistance. The C797S mutation, which occurs at the covalent binding site of osimertinib, prevents irreversible drug binding to the EGFR kinase domain. Configuration analysis demonstrated that 13 patients (65.0%) harbored C797S and T790M mutations in cis configuration, meaning both mutations resided on the same EGFR allele. In contrast, six patients (30.0%) exhibited trans configuration, where C797S and T790M mutations were present on different alleles. One patient (5.0%) showed heterogeneous tumor populations with both cis and trans configurations detected in different metastatic sites. The cis configuration is particularly significant as it confers complete resistance to osimertinib while maintaining resistance to first-generation TKIs, thereby eliminating options for EGFR-directed therapy. Patients with C797S mutations in trans configuration theoretically retain sensitivity to combined EGFR TKI strategies. The median time to development of C797S mutation was 13.8 months after osimertinib initiation, and these patients demonstrated a median overall survival of 19.6 months from confirmed progression on osimertinib, which was intermediate compared to other resistance mechanisms (Figure 1).

### MET amplification and bypass signaling activation

MET amplification represented the second most common resistance mechanism, identified in 13 patients (15.1%). Quantitative assessment revealed high-level MET amplification with a median MET/CEP7 ratio of 5.2 (range: 2.3–12.4) by fluorescence *in situ* hybridization. To elucidate the functional role of MET amplification in osimertinib resistance, we performed *in vitro* validation experiments using EGFR-mutant cell lines. PC9 cells engineered to overexpress MET or treated with hepatocyte growth factor demonstrated a 45-fold increase in osimertinib IC50 compared to parental cells (IC50: 4.5 μM versus 0.1 μM, P < 0.001; mean ± SD from three independent biological replicates). Western blot analysis, quantified by densitometric analysis (normalized to GAPDH) across three independent replicates, confirmed that MET amplification mediated resistance through bypass activation of downstream signaling pathways, with sustained phosphorylation of ERK1/2 and AKT observed even in the presence of osimertinib at concentrations sufficient to inhibit EGFR phosphorylation. Combination treatment with osimertinib and the MET inhibitor capmatinib effectively suppressed both ERK and AKT phosphorylation and restored sensitivity to EGFR inhibition. Clinically, patients with MET amplification-mediated resistance had a median overall survival of 16.8 months after progression, and three patients who received subsequent MET inhibitor-based therapy showed partial responses(Table 3).

**TABLE 3:** MET amplification-mediated osimertinib resistance.

Category	Parameter	Results	Statistical significance
Clinical prevalence	Patients with MET amplification	13/86 (15.1%)	Not applicable
Molecular characterization	MET/CEP7 ratio by FISH	Median: 5.2 (range: 2.3–12.4)	High level amplification
*In Vitro* validation (PC9 cells)	Osimertinib IC50 parental cells	0.1 μM	Not applicable
	Osimertinib IC50 MET overexpressing cells	4.5 μM	P < 0.001
	Fold change in IC50	45-Fold increase	P < 0.001
Signaling pathway analysis	ERK1/2 phosphorylation	Sustained despite EGFR inhibition	Bypass mechanism confirmed
	AKT phosphorylation	Sustained despite EGFR inhibition	Bypass mechanism confirmed
	EGFR phosphorylation	Inhibited by osimertinib	Expected on target effect
Combination therapy (In vitro)	Osimertinib plus capmatinib on ERK	Complete suppression	Restored sensitivity
	Osimertinib plus capmatinib on AKT	Complete suppression	Restored sensitivity
Clinical outcomes	Median overall survival post progression	16.8 months	Not applicable
	Patients receiving MET inhibitor therapy	3 patients	Not applicable
	Response to MET inhibitor based therapy	Partial responses (all 3 patients)	Clinical benefit demonstrated

Key Findings: MET amplification is a significant resistance mechanism occurring in 15.1% of osimertinib resistant patients. Resistance is mediated through bypass activation of ERK and AKT signaling pathways. Combination therapy with MET inhibitors restores osimertinib sensitivity *in vitro*. Clinical responses to MET inhibitor based therapy validate the therapeutic strategy.

### HER2 amplification and PIK3CA mutations as resistance mechanisms

HER2 amplification was identified in seven patients (8.1%) as an alternative bypass pathway mechanism of resistance. The median HER2/CEP17 ratio was 4.8 (range: 2.5–9.2), indicating high-level amplification. Immunohistochemistry confirmed strong HER2 protein overexpression (3+ intensity) in all cases with amplification. HER2 amplification enables continued signaling through PI3K/AKT and MAPK pathways despite EGFR inhibition, providing an escape route for tumor cells. These patients had relatively poor outcomes, with a median overall survival of 14.2 months from confirmed progression on osimertinib. PIK3CA mutations, which directly activate the PI3K/AKT signaling pathway, were detected in six patients (7.0%). The most common PIK3CA mutations were E545K (3 patients) and H1047R (2 patients), both well-established activating mutations. One patient harbored a rare E542K mutation. These mutations conferred resistance by maintaining downstream signaling activation independent of EGFR status. Interestingly, two patients with PIK3CA mutations also showed concurrent EGFR C797S mutations, suggesting that these mechanisms can coexist and potentially synergize to promote resistance. The median overall survival for patients with PIK3CA mutations was 15.9 months from confirmed progression on osimertinib. Both HER2 amplification and PIK3CA mutations represent potential therapeutic targets for combination or sequential treatment strategies in the post-osimertinib setting (Table 4).

**TABLE 4:** HER2 amplification and PIK3CA mutations as resistance mechanisms: Comprehensive data summary.

Category	Parameter	Results	Additional information
HER2 amplification prevalence	Patients with HER2 amplification	7/86 (8.1%)	Alternative bypass pathway mechanism
HER2 molecular characterization	HER2/CEP17 ratio by FISH	Median: 4.8 (range: 2.5–9.2)	High level amplification
	Immunohistochemistry intensity	3 plus intensity (strong overexpression)	All cases with amplification confirmed
HER2 signaling pathways	PI3K/AKT pathway	Continued signaling despite EGFR inhibition	Escape mechanism
	MAPK pathway	Continued signaling despite EGFR inhibition	Escape mechanism
HER2 clinical outcomes	Median overall survival post resistance	14.2 months	Relatively poor outcomes
PIK3CA mutations prevalence	Patients with PIK3CA mutations	6/86 (7.0%)	Direct PI3K/AKT pathway activation
PIK3CA mutation spectrum	E545K mutation	3 patients	Well established activating mutation
	H1047R mutation	2 patients	Well established activating mutation
	E542K mutation	1 patient	Rare mutation
PIK3CA resistance mechanism	Downstream signaling activation	Independent of EGFR status	Maintains pathway activation
Concurrent mutations	PIK3CA plus EGFR C797S	2 patients	Coexisting mechanisms with potential synergy
PIK3CA clinical outcomes	Median overall survival post progression	15.9 months	Moderate survival outcomes
Therapeutic implications	Treatment strategy potential	Combination or sequential therapy	Both mechanisms are targetable
	Clinical setting	Post osimertinib treatment	Potential therapeutic targets

Key Findings: HER2 amplification occurs in 8.1% of osimertinib resistant patients and serves as a bypass pathway mechanism through continued PI3K/AKT and MAPK signaling. PIK3CA mutations are detected in 7.0% of patients and directly activate downstream signaling independent of EGFR status. The most common PIK3CA mutations are E545K and H1047R, both well characterized activating mutations. Concurrent mutations can coexist, with two patients harboring both PIK3CA and EGFR C797S mutations, suggesting potential synergistic resistance mechanisms. Both HER2 amplification and PIK3CA mutations represent actionable therapeutic targets for future combination or sequential treatment strategies in the post osimertinib setting.

### Histological transformation: small cell transformation and epithelial-mesenchymal transition

Histological transformation emerged as a clinically significant resistance mechanism in 19 patients (22.1% of the cohort), encompassing both small cell lung cancer transformation and epithelial-mesenchymal transition. Small cell lung cancer transformation was confirmed in eight patients (9.3%) through characteristic histological features and immunohistochemical profiling. These transformed tumors exhibited typical small cell morphology with high nuclear-to-cytoplasmic ratio, finely dispersed chromatin, and frequent mitotic figures. Immunohistochemistry demonstrated strong expression of neuroendocrine markers including synaptophysin (8/8 cases), chromogranin A (6/8 cases), and CD56 (7/8 cases), while TTF-1 expression was either lost or markedly diminished. Notably, EGFR mutations including the original sensitizing mutation and T790M were retained in the transformed components, confirming clonal evolution from the original adenocarcinoma. Patients with small cell transformation had the worst prognosis among all resistance mechanisms, with a median overall survival of only 8.3 months from confirmed progression on osimertinib. Epithelial-mesenchymal transition was detected in 11 patients (12.8%) and characterized by morphological changes toward spindle cell or sarcomatoid features. Immunohistochemical analysis revealed loss of epithelial marker E-cadherin in 10 of 11 cases and acquisition of mesenchymal marker vimentin in all cases. These patients demonstrated a median overall survival of 13.7 months from confirmed progression on osimertinib. Both transformation mechanisms represent fundamental changes in tumor biology that require distinct therapeutic approaches, with small cell transformation typically managed with platinum-etoposide chemotherapy rather than continued targeted therapy (Table 5).

**TABLE 5:** Histological transformation: Small cell transformation and epithelial-mesenchymal transition.

Category	Parameter	Results	Additional information
Overall histological transformation	Total patients with transformation	19/86 (22.1%)	Clinically significant resistance mechanism
	Transformation subtypes	SCLC transformation and EMT	Distinct biological processes
SCLC transformation prevalence	Patients with SCLC transformation	8/86 (9.3%)	Confirmed by histology and IHC
SCLC morphological features	Nuclear to cytoplasmic ratio	High	Typical small cell morphology
	Chromatin pattern	Finely dispersed	Characteristic feature
	Mitotic activity	Frequent mitotic figures	High proliferation rate
SCLC immunohistochemistry	Synaptophysin expression	8/8 cases (100%)	Strong neuroendocrine marker
	Chromogranin A expression	6/8 cases (75%)	Neuroendocrine marker
	CD56 expression	7/8 cases (87.5%)	Neuroendocrine marker
	TTF1 expression	Lost or markedly diminished	Loss of adenocarcinoma marker
SCLC molecular features	EGFR mutation retention	Retained in transformed components	Original sensitizing mutation and T790M
	Clonal relationship	Clonal evolution from adenocarcinoma	Confirms transformation origin
SCLC clinical outcomes	Median overall survival post transformation	8.3 months	Worst prognosis among all mechanisms
SCLC treatment approach	Recommended therapy	Platinum etoposide chemotherapy	Not continued targeted therapy
EMT prevalence	Patients with EMT	11/86 (12.8%)	Morphological transformation
EMT morphological features	Histological changes	Spindle cell or sarcomatoid features	Loss of epithelial architecture
EMT immunohistochemistry	E cadherin loss	10/11 cases (90.9%)	Loss of epithelial marker
	Vimentin acquisition	11/11 cases (100%)	Gain of mesenchymal marker
EMT clinical outcomes	Median overall survival post resistance	13.7 months	Moderate survival outcomes
Therapeutic implications	Treatment strategy	Distinct approaches required	Fundamental changes in tumor biology
	Clinical management	Different from targeted therapy	Transformation specific therapy

Key Findings: Histological transformation represents a clinically significant resistance mechanism occurring in 22.1% of osimertinib resistant patients. Small cell lung cancer transformation occurs in 9.3% of patients and is characterized by typical neuroendocrine features with strong expression of synaptophysin, chromogranin A, and CD56, while TTF1 expression is lost or diminished. EGFR mutations are retained in transformed components, confirming clonal evolution from the original adenocarcinoma. SCLC transformation carries the worst prognosis with a median overall survival of only 8.3 months and requires platinum etoposide chemotherapy rather than continued targeted therapy. Epithelial mesenchymal transition is detected in 12.8% of patients and is characterized by loss of E cadherin and acquisition of vimentin, with morphological changes toward spindle cell or sarcomatoid features and a median overall survival of 13.7 months. Both transformation mechanisms represent fundamental changes in tumor biology requiring distinct therapeutic approaches.

### Multiple concurrent resistance mechanisms and prognostic implications

**FIGURE 2:**
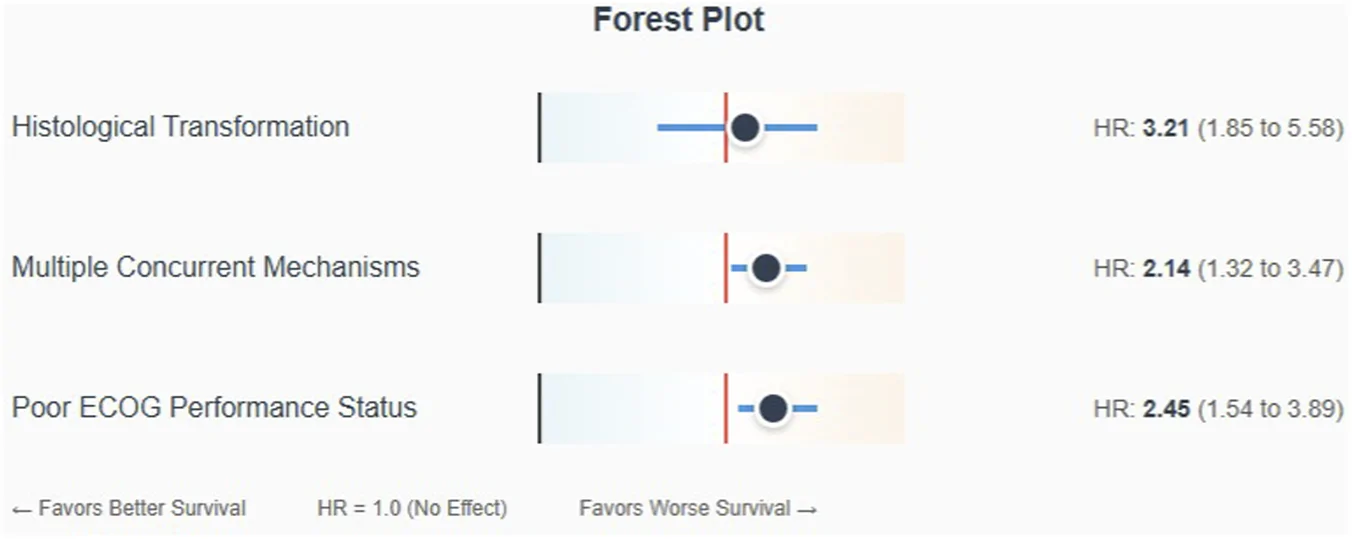
Multivariable Cox regression analysis identifying independent prognostic factors for overall survival after osimertinib resistance. Forest plot showing hazard ratios (HR) and 95% confidence intervals (CI). The vertical line at HR = 1.0 represents no effect. All three factors (histological transformation, multiple concurrent mechanisms, and poor ECOG performance status) were significantly associated with worse survival (all P < 0.01).

A substantial proportion of patients (21 patients, 24.4%) harbored multiple concurrent resistance mechanisms, reflecting the polyclonal nature of acquired resistance and intratumoral heterogeneity (co-occurrence patterns are illustrated in Supplementary Figure S2). The most common combination was EGFR C797S mutation coexisting with MET amplification (6 patients), followed by PIK3CA mutations with bypass pathway alterations (4 patients). Three patients demonstrated triple resistance mechanisms involving EGFR C797S, MET amplification, and additional PIK3CA mutations, representing highly complex resistance patterns. Spatial heterogeneity was evident in eight patients who had multiple sites biopsied, with different resistance mechanisms identified in different metastatic lesions. For instance, one patient showed MET amplification in a liver metastasis while harboring EGFR C797S in a lung lesion. Temporal heterogeneity was also observed, with sequential biopsies in five patients revealing evolution of resistance mechanisms over time. Survival analysis demonstrated that patients with multiple concurrent mechanisms had significantly worse outcomes compared to those with single mechanisms (median overall survival: 11.4 months versus 18.7 months, P = 0.003). Multivariable Cox regression analysis identified histological transformation (hazard ratio: 3.21, 95% CI: 1.85–5.58, P < 0.001), multiple concurrent mechanisms (hazard ratio: 2.14, 95% CI: 1.32–3.47, P = 0.002), and poor ECOG performance status (hazard ratio: 2.45, 95% CI: 1.54–3.89, P < 0.001) as independent adverse prognostic factors for overall survival after osimertinib resistance. These findings emphasize the importance of comprehensive molecular profiling and the potential need for multi-targeted therapeutic strategies to address the complexity of resistance mechanisms in individual patients. To address potential confounding by differential post-progression treatment, a pre-specified sensitivity analysis was conducted restricted to patients who did not receive any subsequent targeted therapy (n = 47). In this subgroup, the survival hierarchy across resistance mechanism categories remained directionally consistent with the primary analysis: patients with SCLC transformation continued to demonstrate the shortest survival (median OS: 7.9 months), while those with single C797S-only resistance showed comparatively longer survival (median OS: 20.1 months), confirming that the observed survival differences reflect intrinsic biological differences rather than treatment heterogeneity alone. Post-progression treatment regimens by resistance subgroup are summarized as follows: patients with SCLC transformation (n = 8) uniformly received platinum-etoposide chemotherapy; patients with MET amplification (n = 13) received capmatinib-based combination therapy (n = 3) or chemotherapy (n = 10); patients with HER2 amplification (n = 7) received anti-HER2 therapy (n = 2) or chemotherapy (n = 5); and patients with C797S mutation (n = 20) received investigational allosteric EGFR inhibitors (n = 4), combination TKI strategies (n = 6), or chemotherapy (n = 10). Patients with unknown resistance mechanisms (n = 9) received best supportive care or chemotherapy per physician discretion (Figure 2).

## Discussion

This comprehensive study systematically characterized the molecular landscape of acquired resistance to osimertinib in 86 patients with EGFR T790M-positive lung adenocarcinoma. Our findings reveal remarkable heterogeneity in resistance mechanisms, with EGFR C797S mutation, bypass pathway activation through MET and HER2 amplification, PIK3CA mutations, and histological transformation representing the predominant mechanisms. The high prevalence of multiple concurrent resistance mechanisms (24.4%) underscores the polyclonal evolution and adaptive capacity of tumors under selective pressure from targeted therapy. Several features distinguish this study from prior reports. The frequency distribution observed here—where bypass pathway activation collectively accounts for 30.2% of resistance events—aligns with and extends recent findings from clinical trial long-term follow-up analyses that confirmed MET amplification and HER2 amplification as increasingly prevalent resistance mechanisms (Papadimitrak et al., 2020). The FLAURA long-term follow-up data similarly demonstrated that bypass pathway mechanisms represent a major acquired resistance category when osimertinib is used in the first-line setting (Ramalingam et al., 2020). Furthermore, the role of HER3/ERBB3-mediated signaling as a bypass mechanism in EGFR TKI resistance, as demonstrated by (Engelman et al., 2007), highlights additional pathways that deserve prospective investigation in future studies. The characterization of spatial and temporal resistance heterogeneity in our cohort underscores the need for comprehensive, multi-site, and longitudinal molecular profiling strategies advocated by recent guidelines. EGFR C797S mutation emerged as the most frequent resistance mechanism, accounting for 23.3% of cases in our cohort. This tertiary mutation occurs at the cysteine residue that forms a covalent bond with osimertinib, thereby preventing irreversible drug binding while preserving kinase activity. Our configuration analysis revealed that 65.0% of C797S mutations existed in cis with T790M, rendering tumors resistant to both osimertinib and first-generation EGFR TKIs. This finding has critical therapeutic implications, as patients with cis-configuration mutations have limited options for subsequent EGFR-directed therapy. In contrast, the 30.0% of patients with trans-configuration mutations theoretically retain sensitivity to combination strategies using first- and third-generation TKIs, though clinical validation of this approach remains limited. The frequency of C797S mutation in our study is consistent with some prior reports but notably lower than the 40%–50% observed in earlier studies. This discrepancy may reflect differences in detection methodologies, timing of resistance biopsy, and patient populations. The relatively lower prevalence in our cohort suggests that alternative resistance mechanisms play increasingly important roles in real-world clinical practice. The intermediate survival outcomes (median OS: 19.6 months) observed in C797S-positive patients indicate that while this mechanism confers therapeutic resistance, it may not fundamentally alter tumor biology as drastically as histological transformation. Bypass pathway activation through MET amplification (15.1%), HER2 amplification (8.1%), and PIK3CA mutations (7.0%) collectively represents a substantial proportion of resistance mechanisms. These alterations enable tumors to maintain proliferative signaling despite effective EGFR inhibition, essentially rewiring cellular signaling networks to circumvent dependency on EGFR. MET amplification-mediated resistance was particularly well-characterized in our study through *in vitro* validation experiments (Wu et al., 2018), which are presented as a focused proof-of-principle for MET-driven bypass signaling rather than as comprehensive functional validation of all identified resistance mechanisms. It is acknowledged that HER2 amplification (8.1%) and PIK3CA mutations (7.0%), while clinically significant and well-supported by external literature, lack direct experimental characterization in the present study; functional validation of these pathways in relevant cell-line models represents an important priority for future investigation. The 45-fold increase in osimertinib IC50 in MET-overexpressing cells, coupled with sustained ERK and AKT phosphorylation despite EGFR inhibition, provides mechanistic evidence for MET-driven bypass signaling (Bean et al., 2007). These *in vitro* findings were corroborated by clinical responses observed in three patients who received MET inhibitor-based therapy, supporting the therapeutic potential of dual EGFR/MET blockade in this molecular subset (Yu et al., 2018). HER2 amplification represents another well-established bypass mechanism that activates overlapping downstream pathways including PI3K/AKT and MAPK signaling (Takezawa et al., 2012; Hsu et al., 2020). The high-level amplification observed in our cohort (median HER2/CEP17 ratio: 4.8) with corresponding 3+ protein overexpression suggests these tumors may be amenable to HER2-directed therapies. The relatively poor survival outcomes (median OS: 14.2 months) in HER2-amplified patients highlight the aggressive nature of this resistance mechanism and the urgent need for effective combination strategies (Takezawa et al., 2012). PIK3CA mutations, particularly the hotspot mutations E545K and H1047R, directly activate PI3K/AKT signaling independent of upstream receptor tyrosine kinases. The observation that two patients harbored concurrent PIK3CA and EGFR C797S mutations suggests potential synergistic effects of multiple resistance mechanisms within individual tumors (Chmielecki et al., 2023). This co-occurrence pattern emphasizes the complexity of resistance evolution and the potential need for multi-targeted therapeutic approaches. Less common resistance mechanisms such as BRAF V600E mutation, identified in 3.5% of our cohort, represent potentially actionable alterations; dabrafenib plus trametinib has demonstrated clinical benefit in BRAF V600E-mutant NSCLC (Planchard et al., 2016). Histological transformation emerged as a clinically significant resistance category, affecting 22.1% of patients in our cohort. Small cell lung cancer transformation (9.3%) represents the most dramatic form of phenotypic plasticity, characterized by complete loss of adenocarcinoma features and acquisition of neuroendocrine characteristics (Offin et al., 2019; Marcoux et al., 2019). The retention of original EGFR mutations in transformed components confirms clonal evolution from the original adenocarcinoma rather than development of a separate primary tumor. This finding has important implications for understanding tumor evolution and resistance mechanisms. The dismal prognosis associated with SCLC transformation (median OS: 8.3 months) reflects both the aggressive biological behavior of small cell histology and the fundamental shift away from EGFR-dependent signaling (Niederst et al., 2015). These patients require a complete change in therapeutic strategy, typically transitioning to platinum-etoposide chemotherapy rather than continued EGFR-targeted therapy. The molecular mechanisms driving histological transformation remain incompletely understood but may involve epigenetic reprogramming, loss of tumor suppressors such as RB1 and TP53, and activation of alternative transcriptional programs (Offin et al., 2019). Epithelial-mesenchymal transition (12.8%) represents another form of phenotypic plasticity characterized by loss of epithelial characteristics and acquisition of mesenchymal features. The near-universal acquisition of vimentin expression and frequent loss of E-cadherin in our cohort provides molecular evidence for this phenotypic switch. While EMT-associated resistance conferred better survival outcomes than SCLC transformation (median OS: 13.7 months), these patients still demonstrated significantly worse outcomes than those with purely genetic resistance mechanisms. The substantial proportion of patients harboring multiple concurrent resistance mechanisms (24.4%) reveals the complex, polyclonal nature of acquired resistance (Chabon et al., 2016). The observation of spatial heterogeneity, with different resistance mechanisms identified in distinct metastatic lesions within individual patients, demonstrates that resistance evolution can proceed along divergent pathways in different tumor deposits. This spatial heterogeneity has profound implications for treatment strategies, as targeting a single resistance mechanism may prove insufficient when alternative mechanisms coexist in other disease sites. Temporal heterogeneity, evidenced by evolution of resistance mechanisms over time in patients with sequential biopsies, suggests that resistance is not a static phenomenon but rather continues to evolve under ongoing selective pressure. The significantly worse survival outcomes in patients with multiple concurrent mechanisms (median OS: 11.4 months versus 18.7 months for single mechanisms) highlight the clinical impact of resistance complexity and the challenges in managing these patients (Chabon et al., 2016). Our multivariable Cox regression analysis identified histological transformation, multiple concurrent mechanisms, and poor ECOG performance status as independent adverse prognostic factors. The hazard ratio of 3.21 for histological transformation confirms its devastating clinical impact, while the hazard ratio of 2.14 for multiple concurrent mechanisms quantifies the additive negative effect of resistance complexity. The molecular heterogeneity of osimertinib resistance documented in our study has important implications for clinical management (Oxnard et al., 2018). First, comprehensive molecular profiling at disease progression is essential for identifying specific resistance mechanisms and guiding subsequent therapeutic decisions. Emerging artificial intelligence-assisted decision support tools represent a complementary layer in this framework: large language model-based systems have recently shown promise in supporting real-time clinical decision-making for complex diagnostic scenarios, suggesting that similar AI-driven approaches may eventually facilitate rapid resistance mechanism interpretation and post-progression treatment selection in EGFR-mutant NSCLC (Jiang et al., 2025). The 10.5% of patients in whom no identifiable resistance mechanism was detected despite comprehensive NGS analysis suggests that additional mechanisms remain to be discovered, potentially involving non-coding genomic alterations, epigenetic modifications, or tumor microenvironment factors. Second, the targetable nature of many resistance mechanisms provides opportunities for rational combination or sequential therapy approaches. MET amplification, HER2 amplification, and specific EGFR C797S configurations represent potentially actionable alterations for which targeted agents are available or in clinical development. Early clinical trials of combination EGFR/MET inhibition have shown promising preliminary results, and our data provide additional support for this strategy in appropriately selected patients (Yu et al., 2018).

## Limitations

Several limitations of our study warrant consideration. First, the retrospective design introduces selection bias: of 143 eligible patients who progressed on osimertinib during the study period, 57 (39.9%) could not be included due to unavailability of post-progression molecular profiling, predominantly because of poor ECOG performance status (PS 2–3: 64.9% vs. 41.9% in the included cohort, P = 0.004) or more rapidly progressive disease (median time to progression: 9.8 vs. 14.2 months, P = 0.001). A patient screening flowchart is provided in Supplementary Figure S1. This directional bias implies that reported frequencies of biologically aggressive resistance alterations—particularly SCLC transformation—likely underestimate true prevalence in the overall progressing population. Second, the differential sensitivity of liquid biopsy relative to tissue biopsy for copy number alterations may have led to underdetection of MET and HER2 amplification in the 22.1% of patients who underwent plasma-based profiling; a resistance mechanism was identified in 92.5% of tissue biopsy patients versus 68.4% of liquid biopsy patients (P = 0.002), and MET amplification was detected exclusively in tissue biopsy specimens, consistent with the known limited sensitivity of plasma-based copy number detection. These findings support preferential use of tissue biopsy for post-progression profiling when copy number alterations are clinically suspected. Third, the functional validation experiments employed MET overexpression and HGF stimulation models rather than genuine genomic amplification; while informative for establishing mechanistic proof-of-principle, these models do not fully recapitulate the transcriptional and chromosomal complexity of clinical MET amplification. Fourth, survival analyses for resistance subgroups with fewer than five patients were not performed given insufficient statistical power. Fifth, the single-institution nature of this study may limit generalizability to populations with different ethnic backgrounds, treatment patterns, or access to comprehensive molecular profiling.

## Conclusion

This comprehensive molecular characterization of osimertinib resistance in EGFR T790M-positive lung adenocarcinoma reveals remarkable heterogeneity encompassing EGFR tertiary mutations, bypass pathway activation, and histological transformation. The high prevalence of multiple concurrent resistance mechanisms (24.4%) underscores the polyclonal evolution of tumors under targeted therapy pressure. Histological transformation, particularly small cell lung cancer transformation, confers the worst prognosis and necessitates a fundamental shift in therapeutic strategy. Comprehensive post-progression molecular profiling is essential for identifying specific resistance mechanisms and guiding individualized treatment decisions. Rational combination strategies targeting bypass signaling pathways, such as dual EGFR/MET inhibition, hold promise for improving outcomes in appropriately selected patients. Future prospective studies with larger cohorts and independent validation are warranted to further characterize resistance heterogeneity and optimize post-progression therapeutic strategies in EGFR T790M-positive lung adenocarcinoma.

## Data Availability

The original contributions presented in the study are publicly available. This data can be found at Figshare with the doi 10.6084/m9.figshare.33214578, at https://doi.org/10.6084/m9.figshare.33214578.
