# Supplementary material for: Mechanisms of acquired resistance to osimertinib in EGFR T790M-Positive lung adenocarcinoma
Source: Front Genet. 2026 Sep 4;17:1827187. doi: 10.3389/fgene.2026.1827187 (PMC13585410; doi:10.3389/fgene.2026.1827187)
Supplement: Supplementary file 1 [file DataSheet1.docx]

**Supplementary Materials**

**Mechanisms of Acquired Resistance to Osimertinib in EGFR T790M-Positive Lung Adenocarcinoma**

*Kefan Cui, Renjie Zhu, Jingmeng Li*

**Contents**

Figure S1	Patient Screening Flowchart

Table S1	Resistance Mechanism Detection Rates Stratified by Biopsy Type

Table S2	Concurrent Resistance Mechanism Combinations

Table S3	Site-Specific Resistance Mechanisms in Multi-site Biopsy Cases

**Figure S1. Patient Screening Flowchart**

The figure below illustrates the patient screening and selection process. Of 143 patients with EGFR T790M-positive lung adenocarcinoma who progressed on osimertinib during the study period (January 2018 – December 2023), 86 (60.1%) were included in the final analysis.

**EGFR T790M-positive lung adenocarcinoma patients who progressed on osimertinib**

**n = 143**

*January 2018 – December 2023*

**▼**

**Assessed for molecular profiling at progression**

**n = 143**

*Tissue biopsy attempted or liquid biopsy collected*

**▼**

**Excluded (n = 57, 39.9%):**

• Insufficient tumor material / failed QC (n = 24)

• ECOG PS 2–3 / too unwell for biopsy (n = 19)

• Patient/clinician declined biopsy (n = 9)

• Rapidly progressive disease; died before biopsy (n = 5)

Excluded patients vs. included patients:

– ECOG PS 2–3: 64.9% vs. 41.9% (P = 0.004)

– Median time to progression: 9.8 vs. 14.2 months (P = 0.001)

**▼**

**Final analysis cohort**

**n = 86 (60.1%)**

*Tissue biopsy: 67 (77.9%) | Liquid biopsy: 19 (22.1%)*

**▼**

**Successful molecular profiling → resistance mechanism identified in 77/86 (89.5%)**

*Abbreviations: ECOG PS, Eastern Cooperative Oncology Group performance status; QC, quality control. *P-values calculated using chi-square test or Mann–Whitney U test, as appropriate.*

**Table S1. Resistance Mechanism Detection Rates Stratified by Biopsy Type**

Resistance mechanisms were identified at a significantly higher rate in patients undergoing tissue biopsy than in those undergoing liquid biopsy (92.5% vs. 68.4%, P = 0.002). Notably, MET amplification was exclusively detected in tissue biopsy specimens, consistent with the known lower sensitivity of plasma-based cell-free DNA assays for copy number alterations. The proportion of patients with no identifiable mechanism was significantly higher in the liquid biopsy group (31.6% vs. 7.5%, P = 0.012).

| **Resistance Mechanism** | **Tissue Biopsy (n=67)** | **Liquid Biopsy (n=19)** | **P-value** |
| --- | --- | --- | --- |
| **Any mechanism identified** | 62/67 (92.5%) | 13/19 (68.4%) | **0.002*** |
| EGFR C797S mutation | 17/67 (25.4%) | 3/19 (15.8%) | 0.381 |
| MET amplification | 13/67 (19.4%) | 0/19 (0.0%) | **0.049*** |
| HER2 amplification | 7/67 (10.4%) | 0/19 (0.0%) | 0.196 |
| PIK3CA mutation | 5/67 (7.5%) | 1/19 (5.3%) | 1.000 |
| SCLC transformation | 7/67 (10.4%) | 1/19 (5.3%) | 0.668 |
| EMT | 9/67 (13.4%) | 2/19 (10.5%) | 1.000 |
| Multiple concurrent mechanisms | 18/67 (26.9%) | 3/19 (15.8%) | 0.374 |
| **No identifiable mechanism** | **5/67 (7.5%)** | **6/19 (31.6%)** | **0.012*** |

*P-values calculated using Fisher's exact test. *Statistically significant (P < 0.05). Abbreviations: SCLC, small-cell lung cancer; EMT, epithelial-mesenchymal transition.*

*Note: Individual mechanism counts do not sum to total due to patients with multiple concurrent mechanisms.*

**Table S2. Concurrent Resistance Mechanism Combinations (n = 21 patients)**

Twenty-one patients (24.4%) harbored two or more concurrent resistance mechanisms at progression. Concurrent mechanisms were defined as two or more distinct resistance alterations detected in the same patient at progression, regardless of biopsy site. The most prevalent combination was EGFR C797S with MET amplification (n = 6, 28.6%).

| **Resistance Mechanism Combination** | **Number of Patients (n=21)** | **Percentage (%)** |
| --- | --- | --- |
| EGFR C797S + MET amplification | 6 | 28.6 |
| PIK3CA mutation + EGFR C797S | 4 | 19.0 |
| EGFR C797S + HER2 amplification | 3 | 14.3 |
| MET amplification + PIK3CA mutation | 3 | 14.3 |
| EGFR C797S + MET amplification + PIK3CA mutation | 3 | 14.3 |
| Other combinations | 2 | 9.5 |

*This definition does not formally distinguish clonal from subclonal events. Exploratory cancer cell fraction estimation using variant allele frequency data, where available, is presented in Supplementary Figure S2 (not shown).*

**Table S3. Site-Specific Resistance Mechanisms in Multi-site Biopsy Cases (n = 8 patients)**

Eight patients underwent biopsy of two or more anatomically distinct tumor sites at progression. Different resistance mechanisms were identified at different sites in all eight cases, demonstrating spatial heterogeneity of resistance evolution. These findings highlight the potential for single-site biopsy to incompletely characterize the full spectrum of resistance mechanisms in a given patient.

| **Patient** | **Biopsy Site 1** | **Mechanism (Site 1)** | **Biopsy Site 2** | **Mechanism (Site 2)** |
| --- | --- | --- | --- | --- |
| 1 | Liver metastasis | MET amplification | Lung (primary) | EGFR C797S |
| 2 | Pleural effusion | EMT | Adrenal metastasis | SCLC transformation |
| 3 | Bone metastasis | HER2 amplification | Lung (primary) | PIK3CA E545K |
| 4 | Brain metastasis | EGFR C797S (cis) | Liver metastasis | MET amplification |
| 5 | Lymph node | PIK3CA H1047R | Lung (primary) | EGFR C797S (trans) |
| 6 | Adrenal metastasis | SCLC transformation | Lung (primary) | EGFR C797S + MET amplification |
| 7 | Pleural effusion | EMT | Lung (primary) | HER2 amplification |
| 8 | Bone metastasis | EGFR C797S (cis) | Lymph node | MET amplification + PIK3CA mutation |

*Abbreviations: SCLC, small-cell lung cancer; EMT, epithelial-mesenchymal transition; cis, C797S and T790M on the same allele; trans, C797S and T790M on different alleles.*

*All supplementary materials correspond to analyses referenced in the revised manuscript. These data are provided to support reproducibility and to address reviewer concerns regarding selection bias (Figure S1), biopsy-type detection sensitivity (Table S1), resistance complexity (Table S2), and spatial heterogeneity (Table S3).*
